# Step-by-step reaction-powered mechanical motion triggered by a chemical fuel pulse†
†Electronic supplementary information (ESI) available. See DOI: 10.1039/c8sc05469j


**DOI:** 10.1039/c8sc05469j

**Published:** 2019-01-04

**Authors:** Qiang Shi, Chuan-Feng Chen

**Affiliations:** a Beijing National Laboratory for Molecular Science , CAS Key Laboratory of Molecular Recognition and Function , Institute of Chemistry , Chinese Academy of Sciences , Beijing 100190 , China . Email: cchen@iccas.ac.cn; b University of Chinese Academy of Sciences , Beijing 100049 , China

## Abstract

Natural molecular machines perform important tasks in organisms under a reasonable energy supply strategy: a series of step-by-step biochemical reactions after the intake of a fuel such as glucose. As analogues of biomolecular machines, most artificial molecular machines and shuttles are still powered by two opposite stimuli, such as acid and base or oxidation and reduction. This inconvenient stimulus method for artificial molecular machines and shuttles creates great obstacles for realizing more functions of artificial molecular machines and shuttles owing to low energy utilization efficiency and troublesome operation. In this work, we report a new step-by-step reaction system combining two known reactions: (1) the formation of [bis(trifluoroacetoxy)iodo]benzene from the reaction of iodosylbenzene and trifluoroacetic acid; and (2) the catalytic oxidation of alcohols by [bis(trifluoroacetoxy)iodo]benzene in the presence of TEMPO. Owing to the acid absorption features of the first reaction and the acid releasing characteristics of the second reaction, the new step-by-step reaction obtains a pH oscillation feature. Taking advantage of the pH oscillation feature, the new reaction cycle triggered by iodosylbenzene was coupled with an acid–base switchable helicarene-based molecular shuttle. Benefitting from the reaction rate difference of the two known reactions (the reaction rate of the first reaction is faster than that of the second reaction), the movement of the helicarene on the axle could be controlled automatically by the reaction system through adding iodosylbenzene to a solution of alcohol, TEMPO and protonated rotaxane, and the manual addition of another opposite stimulus could be avoided.

## Abstract 2

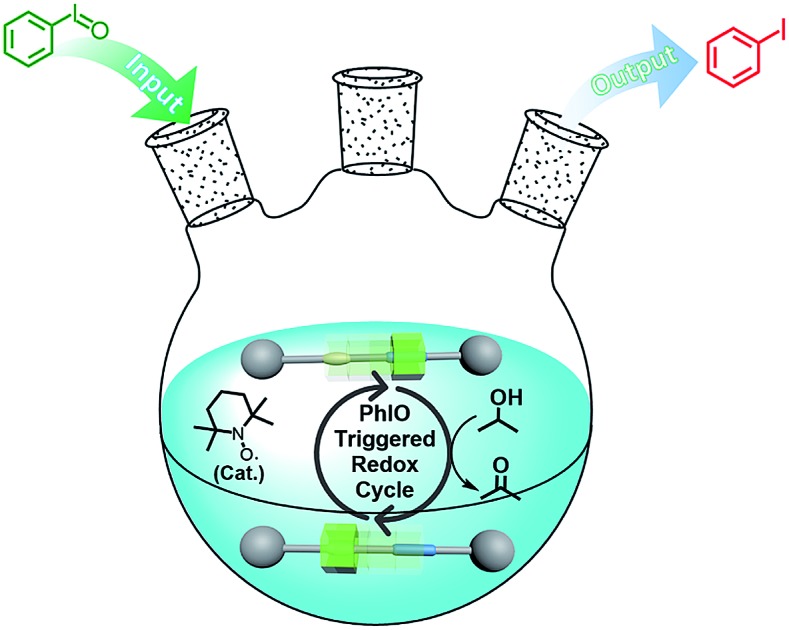
With the discovery of the iodosylbenzene-triggered pH oscillating reaction, the chemically fueled movement of helicarene-based [2]rotaxane could be successfully achieved.

## Introduction

Biomolecular machines are carrying out complex tasks every moment to maintain the functions of our bodies. For example, ATP synthase, which is a kind of protein motor, can synthesize ATP using energy from a proton concentration gradient between the two sides of a membrane,^1^–^4^ and myosin and actin filaments work together to move our muscles.^5^ Inspired by nature, scientists have designed and synthesized a variety of artificial molecular machines^6^–^31^ for the purpose of achieving a goal that is comparable to, or even surpassing, nature's molecular motors. Artificial molecular machines based on mechanically interlocked molecules (MIM) consist of two or more components that can move relatively in response to inputted stimulation.^32^,^33^

How to provide energy to drive the motion of molecular machines is an important issue. In nature, most of the biomolecular machines are powered by the energy from a series of biochemical reactions. For instance, respiration^2^ is one of the main processes to provide energy to organisms. During respiration, glucose is transferred into carbon dioxide and water through glycolysis or the citric acid cycle and the related synthesis and hydrolysis of ATP. At the same time, a lot of energy is released and utilized to drive the motion of biomolecular machines. In these biochemical processes, the initial reactant, such as glucose, usually undergoes continuous multi-step (or step-by-step) reactions to transform into the final products (CO_2_ and H_2_O) and release energy. The energy supply strategy for biomolecular machines allows biomolecular machines to perform more complex functions in organisms. The utilization of step-by-step reactions can improve the energy generation efficiency.^2^ However, compared with the energy supply strategy of biomolecular motors, the strategy of artificial molecular machines or molecular shuttles is more primitive. At present, the operation of most artificial molecular machines or shuttles still follows an inconvenient and “ancient” principle: utilizing one stimulus to propel the machine into a new state, and manually adding an opposite stimulus to reset the system to the original state.^34^–^38^ This inconvenient strategy of energy supply for artificial molecular machines and shuttles limits their further applications in many field. Recently, Stefano's group^19^,^39^,^40^ and Leigh's group^13^ reported a decarboxylation strategy to successfully construct a new kind of molecular machine powered by the decarboxylation of 2-cyano-2-phenylpropanoic acid^41^ or 2,2,2-trichloroacetic acid, respectively. Although the decarboxylation strategy could provide energy efficiently for the movement of artificial molecular machines with specific structures, such examples of mechanical motions powered by step-by-step reactions are still very limited. In particular, how to develop a new step-by-step reaction system to operate the movement of a MIMs-based artificial molecular machine or shuttle like the biomolecules in organisms is still a great challenge.

Previously, we have proved the capabilities of helicarene^42^–^46^ to form host–guest complexes with protonated pyridinium salts, and further constructed an acid–base controllable rotaxane-based molecular switch.^47^ Herein we report a step-by-step reaction system with a pH oscillation feature and the further application of the system into the chemical fuel-driven mechanical motion of a helicarene-based [2]rotaxane.

## Results and discussion

**Scheme 1:**
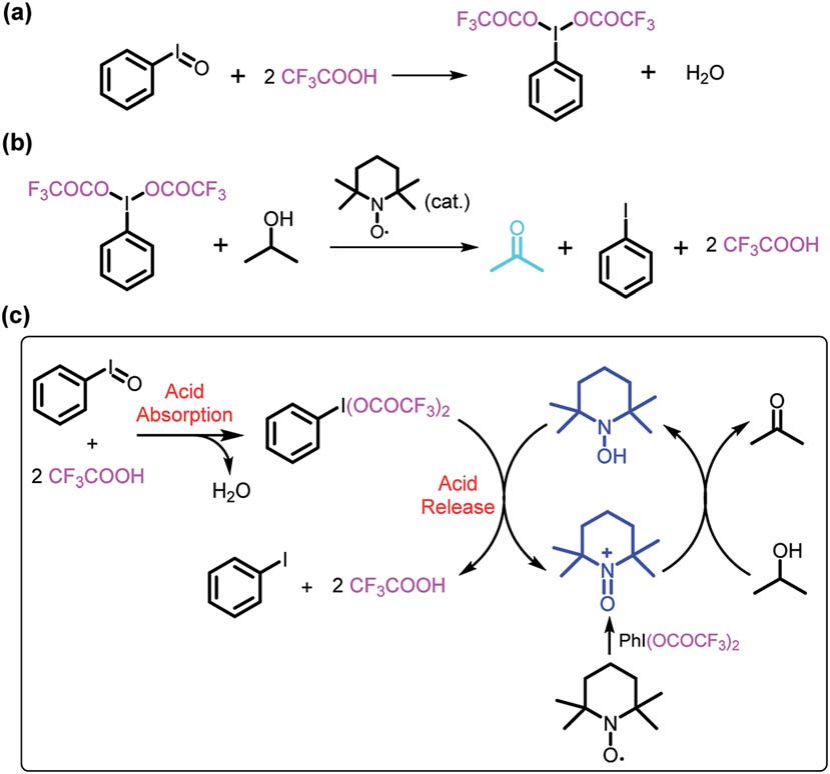
(a) The reaction of PhIO and TFA, (b) the TEMPO-catalyzed oxidation of isopropanol in the presence of BTAIB, and (c) the PhIO-triggered redox cycle with TFA absorption and release (a + b).

The reaction of iodosylbenzene (PhIO)^48^ with trifluoroacetic acid (TFA) formed [bis(trifluoroacetoxy)iodo]benzene (BTAIB, Scheme 1a).^49^,^50^ It was also known that BTAIB could be utilized as a kind of hypervalent iodine reagent to participate in the oxidation of isopropanol (i-PrOH) in the presence of a catalytic amount of TEMPO (Scheme 1b).^51^–^53^ In Scheme 1a, TFA was absorbed. Inversely, TFA could be released from BTAIB after the catalytic oxidation of i-PrOH occurred (Scheme 1b). Because the product in Scheme 1a could be used as the reactant in Scheme 1b, a step-by-step reaction system with pH oscillation property could thus be constructed by coupling the two reactions (Scheme 1c). The addition of PhIO can trigger the cycle of the oscillation system. After PhIO runs out, the cycle stops. This system with the absorption and release of TFA encouraged us to further construct a pH oscillation reaction-powered molecular machine.

The rotaxane **RH**, containing a protonated pyridinium subunit and the alkyl group, with two possible different states was then designed and synthesized (see ESI† for the detailed synthesis and characterization). As expected, the shuttling movement of the macrocycle in the rotaxane could be efficiently controlled by TFA and triethylamine (TEA) (Fig. S15†).

**Fig. 1:**
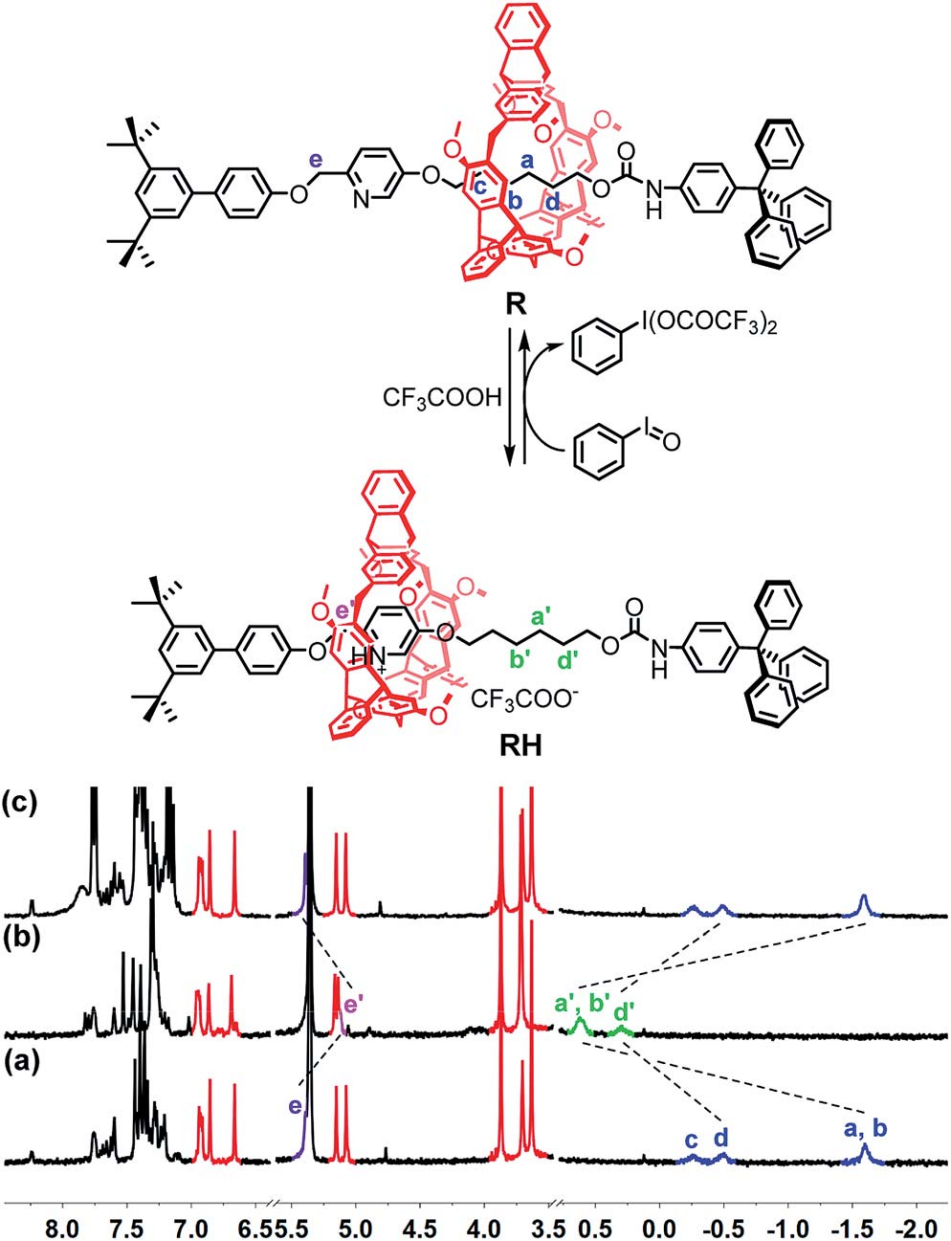
PhIO–TFA controlled switchable movement of **R**, and the ^1^H NMR spectra (300 MHz, CD_2_Cl_2_, 298 K) of (a) **R**; (b) **RH** (**R** + 4 equiv. of TFA); and (c) b + 2.5 equiv. of PhIO.

To verify the feasibility of the pH oscillation reaction-powered motion of the rotaxane triggered by PhIO, the half cycle from **RH** to **R** as shown in Fig. 1 was firstly tested. In the ^1^H NMR spectrum of **R**, the methylene protons H_a_–H_d_ all appeared at upfield of –1.60, –0.51 and –0.27 ppm, respectively, owing to the strong shielding effect of the helicarene (Fig. 1a), suggesting that the macrocycle was inclined to stay at the methylene groups in the neutral state of the rotaxane. After 4 equiv. of TFA was added to **R**, all signals of H_a_–H_d_ shifted to 0.31–1.18 ppm, suggesting that the macrocycle moved from the alkyl group to the pyridinium site to form **RH** (Fig. 1b). After 2.5 equiv. of PhIO was added into the system, TFA reacted with PhIO to produce BTAIB, while the pyridinium in rotaxane **RH** was deprotonated to lose its ability to complex with the helicarene, which resulted in movement of the macrocycle to the alkyl group. Correspondingly, the signals of H_a_–H_d_ were all moved to the upfield (Fig. 1c). Moreover, the reaction rate of this process was relatively fast, and the reaction was completed after PhIO was dissolved.

**Fig. 2:**
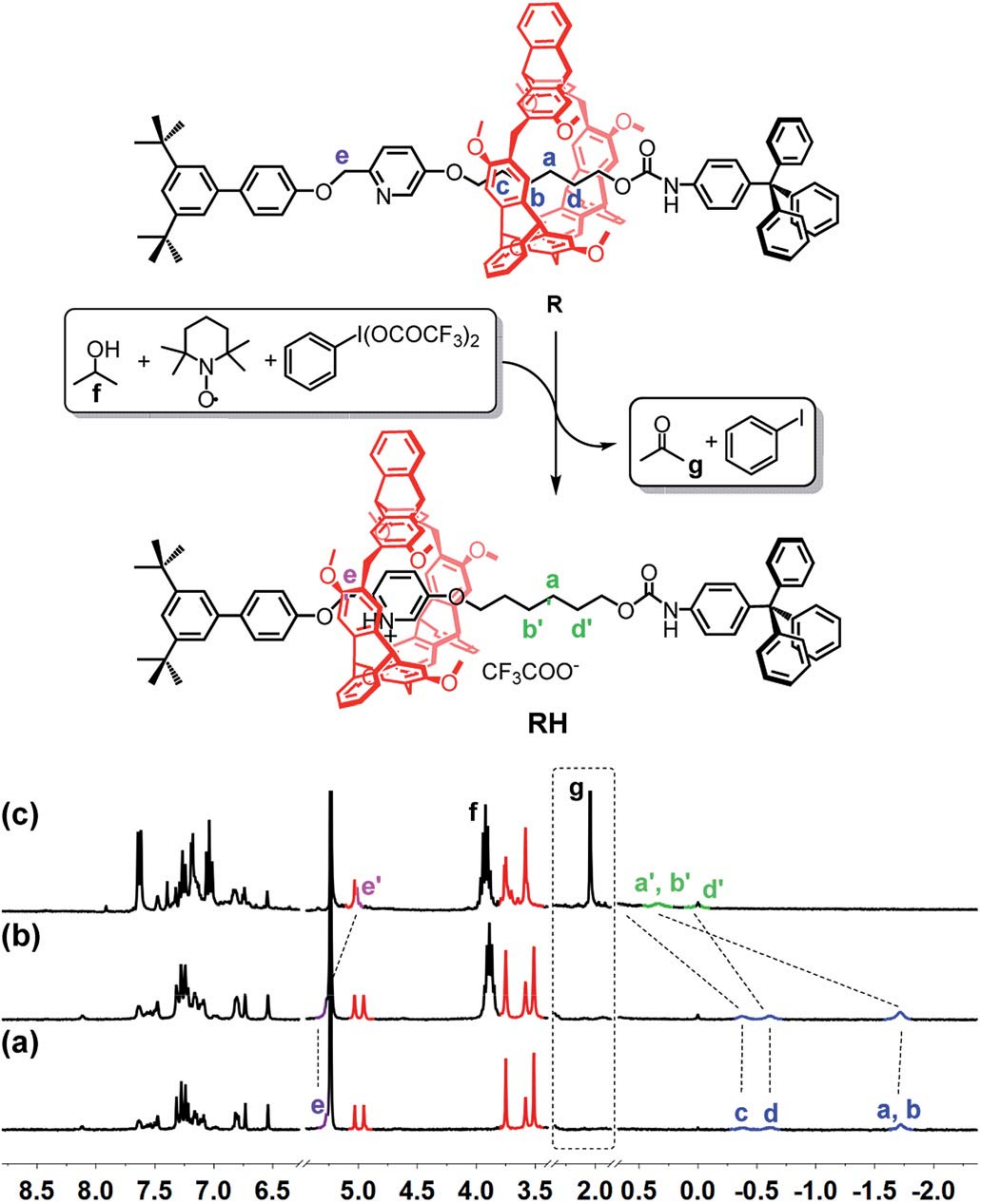
Catalytic oxidation reaction-powered motion of rotaxane and ^1^H NMR spectra (300 MHz, CD_2_Cl_2_, 298 K) of (a) **R**; (b) **R** + 0.5 equiv. of TEMPO + 25 equiv. of i-PrOH; and (c) b + 2 equiv. of BTAIB after 8 h.

The catalytic oxidation powered motion from **R** to **RH** was also investigated (Fig. 2). First, i-PrOH and TEMPO were added into the CD_2_Cl_2_ solution of **R**. In Fig. 2b, the upfield signals of the methylene groups indicated that the rotaxane was in the **R** state. When BTAIB was added into the above solution, the TEMPO-catalyzed oxidation of isopropanol to acetone occurred. After 8 hours, the reaction finished, and the proton signals of the methylene groups in **R** all moved downfield (0.31–1.18 ppm, Fig. 2c), indicating the formation of **RH**. Especially, the emergence of the signal of acetone (H_g_) proved the occurrence of the catalytic oxidation reaction. After the reaction reached equilibrium, about 92% of **R** was changed to **RH**.

Compared with the reaction of PhIO and TFA, the rate of the catalytic oxidation reaction is slow. This feature allows us to further detect the kinetics of the reaction. After 2 equiv. of BTAIB was added to the solution of **R**, 0.5 equiv. of TEMPO and excess i-PrOH in CD_2_Cl_2_, the percentage of **R** state and **RH** state in the [2]rotaxane changed over time. The chemical shifts of protons H_a_ (H_a′_) and H_b_ (H_b′_) were used to detect and calculate the changes of the percentages of **R** and **RH** at different reaction times^10^ (Fig. S16†). The percentage change of **R** to **RH** was very fast in the first two hours, corresponding to the fast reaction rate. The equilibrium was reached slowly in the next 6 hours (Fig. S17†). According to the chemical shifts of H_a_ and H_b_, after the reaction reached equilibrium, about 92% of **R** was changed to **RH**.

**Fig. 3:**
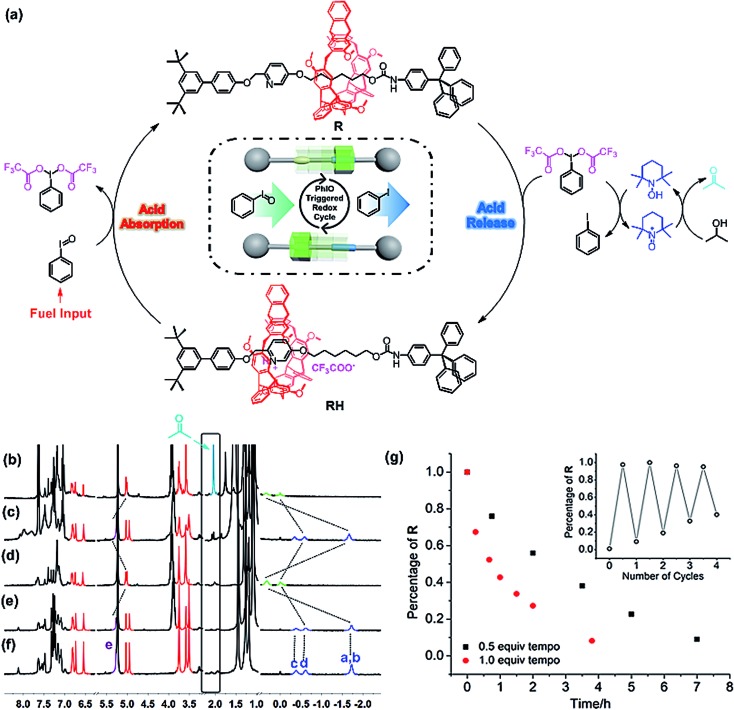
(a) Mechanism and illustration of the chemical fueled switchable motion of the helicarene-based [2]rotaxane powered by PhIO-triggered redox cycle; ^1^H NMR spectra (300 MHz, CD_2_Cl_2_, 298 K) of (f) **R**, (e) **R** + 0.5 equiv. of TEMPO + 25 equiv. of i-PrOH, (d) e + 2 equiv. of TFA, (c) d + 2 equiv. of PhIO, and (b) c after 7 h; and (g) kinetics of the mechanical movement controlled by different concentrations of TEMPO. Insert: reversible motion in the presence of 0.5 equiv. of TEMPO followed by the addition of the chemical fuel.

By combination of the two half cycles proved as above, the reaction-powered motion of [2]rotaxane could thus be realized. The movement process powered by the step-by-step reaction cycle is depicted in Fig. 3a, and the switchable movements were investigated by the ^1^H NMR experiments. First, compared with the ^1^H NMR spectrum of [2]rotaxane **R** in CD_2_Cl_2_ (Fig. 3f), it was found that the presence of TEMPO and i-PrOH resulted in almost no changes of the proton signals of **R** (Fig. 3e), indicating that both TEMPO and i-PrOH did not interact with **R**. After 2 equiv. of TFA was added to the above solution, the pyridine was protonated and the macrocycle moved from the alkyl group to the pyridinium site to form **RH** (Fig. 3d). With the **RH** as the initial state, it was found that upon the addition of the trigger PhIO into the above system, the peaks of protons H_a_–H_d_ all appeared upfield (Fig. 3c), suggesting that PhIO absorbed the TFA to produce BTAIB, while **RH** was deprotonated to make the macrocycle move to the alkyl group to form **R**. Then, the newly produced BTAIB could participate in the TEMPO-catalyzed oxidation of isopropanol to acetone. Thus, with the increase of the reaction time, BTAIB was gradually changed into iodobenzene while TFA was released, which resulted in the formation of **RH** again. Accordingly, all proton signals of [2]rotaxane were back to the original **RH** state after the reaction had proceeded for seven hours (Fig. 3b). Meanwhile, the proton signal of acetone was also observed, which indicated that the back and forth movement of the [2]rotaxane was caused by the PhIO-triggered redox cycle. As mentioned above, the reaction of PhIO and TFA is much faster than the TEMPO mediated oxidation of i-PrOH to acetone by BTAIB. The difference in these two reactions rates is the key factor to drive the normal operation of the process.

The effect of the concentration of TEMPO on the rate of the switchable process was further studied. As shown in Fig. 3g, it was found that when 0.5 equiv. of TEMPO was used, the switchable process took about 7 hours to complete. After the concentration of TEMPO was increased to 1.0 equivalent, the system completed a motion process within 4 hours, which was obviously faster than that of the system with 0.5 equiv. of TEMPO. It was further found that in the two systems with different amounts of TEMPO, about 92% rotaxane **R** could be transferred to the **RH** state. Moreover, the insert graphic in Fig. 3g also shows that PhIO could trigger the reversible motion four times. After four cycles, about 70% of the [2]rotaxane could be recycled under the conditions of sufficient i-PrOH and catalyst activity.

## Conclusions

In conclusion, we have discovered a step-by-step reaction system with the absorption and release of trifluoroacetic acid by a combination of the formation of BTAIB and the subsequently promoted TEMPO-catalyzed oxidation of isopropanol to acetone. Taking advantage of the pH oscillation properties of the reaction, we have successfully achieved the mechanical movement of an acid–base switchable rotaxane by a chemical fuel. This work represents the new pH oscillating chemical fuel powered mechanical movement of an artificial molecular shuttle, and the pH oscillation system could also provide new opportunities for potential applications in molecular machines and functional materials.

## Conflicts of interest

There are no conflicts to declare.

## Supplementary Material

Supplementary information
