# Supplementary material for: Step-by-step reaction-powered mechanical motion triggered by a chemical fuel pulse
Source: Chem Sci. 2019 Jan 4;10(8):2529–33. doi: 10.1039/c8sc05469j (PMC6385870; doi:10.1039/c8sc05469j)
Supplement: Supplementary file 1 [file SC-010-C8SC05469J-s001.pdf]

Electronic Supplementary Material (ESI) for Chemical Science.
This journal is © The Royal Society of Chemistry 2019

Electronic Supplementary Information

A step-by-step reaction powered mechanical motion triggered by a chemical fuel pulse

Qiang Shi[a,b] and Chuan-Feng Chen[a,b,*]

[a]*Beijing National Laboratory for Molecular Sciences, CAS Key Laboratory of Molecular Recognition and Function Institute of Chemistry, Chinese Academy of Sciences, Beijing 100190, China*; [b]*University of Chinese Academy of Sciences, Beijing 100049, China*

Email: cchen@iccas.ac.cn

Table of Contents

1. Materials and methods……………………………………………..…..…..………S2
2. Synthesis of new compounds…………………………………..…..…..……..S2
3. Copies of NMR spectra of new compounds………………………………………S7
4. Acid-base controlled motion of the [2]rotaxane **R**………………..……..……S14
5. The dynamics of the oxidation reaction controlled motion of **R** powered by BTAIB…….……………………………………………………………..……..……..S14
6. The dynamic of the mechanical motion of **R** with different amount of TEMPO……………………………………………………………………....……..S16
7. Repeated chemically fueled motion……………………..……………..……S17

1. Materials and methods

All reactions were carried out under argon using oven-dried glassware. TLCs were performed on silica gel GF254; unless otherwise indicated, all reagents were obtained from commercial sources. Chromatograms were visualized with UV light (254 and 360 nm). Flash column chromatography was performed on 200-300 mesh silica gel. Melting points were determined using WRR melting point apparatus and were uncorrected. Commercial reagents were used without further purification. Anhydrous solvents were dried from 4 Å molecular sieves. ^{1}H NMR spectrawere recorded on the Brucker® Avance III 400 MHz NMR spectrometer and Bruker DMX300 NMR or Bruker® Avance III 500 MHz NMR spectrometer at 298 K. Electrospray ionization mass spectra (ESI-MS) were recorded on the Thermo Fisher® Exactive high-resolution LC-MS spectrometer.

2. Synthesis of New Compounds

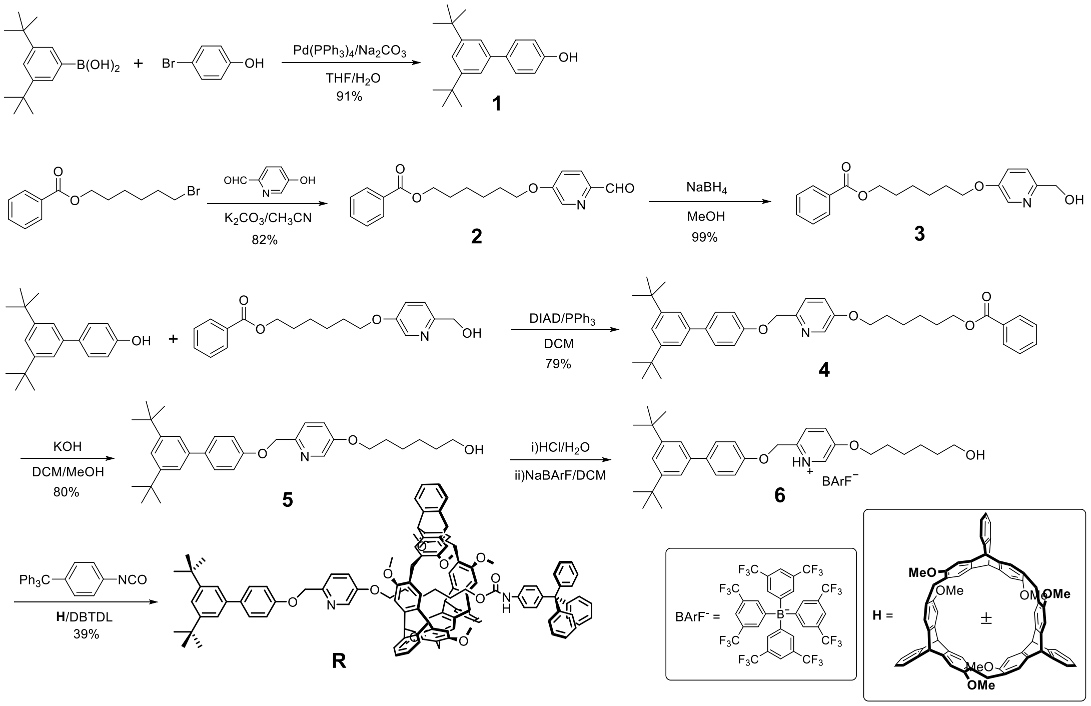

Scheme S1. Synthesis route of **R1**

Synthesis of 1. Na_2CO_3 (13 g, 122.6 mmol) was added in the solution of 3,5-di-tert-butylbenzeneboronic acid (2 g, 8.54 mmol) and *p*-bromophenol

(1.48 g, 8.6 mmol) in THF (100 mL) and distilled water (60 mL). After stirred in argon atmosphere at 70 ℃ for 30 min, Tetrakis(triphenylphosphine)-palladium(0) (200 mg, 0.17 mmol) was added to the mixture. After 7 h, the mixture was cooled down to room temperature, and ethyl acetate was pour dumped into the mixture. The water layer was washed 3 times with ethyl acetate and the organic layer was collected. Then the mixture was concentrated under reduced pressure and purified by flash column chromatography (eluent: 1: 2 petroleum ether and dichloromethane) to afford compound **1** (2.19 g, 7.78 mmol) as a white solid. M. p. 72-75 ℃. ^{1}H NMR (500 MHz, $CDCl_3$): δ 1.37 (s, 18H, CH_3), 6.90 (d, J = 5 Hz, 2H, Ar-H), 7.36 (s, 2H, Ar-H), 7.40 (s, 1H, Ar-H), 7.48 (d, J = 5 Hz, 2H, Ar-H). ^{13}C NMR (125 MHz, $CDCl_3$): δ 31.8, 35.3, 115.8, 121.2, 121.6, 129.0, 135.6, 140.4, 151.3, 155.1. HRMS (APCI) for $C_{20}H_{25}O$, *m/z*, $[M-H]^-$, calculated: 281.1911, found: 281.1903.

Synthesis of 2. 1-Benzoyloxy-6-bromohexane (2 g, 7.02 mmol) and 5-hydroxy-2-pyridinecarboxaldehyde (0.86 g, 7.02 mmol) were dissolved in CH_3CN (35 mL) and then K_2CO_3 (2 g) was added. The mixture was stirred at 80 ℃ for 5 h and cooled to room temperature. The solid in the mixture was filtered and the filtrate was concentrated under reduced pressure. The crude product was purified by flash column chromatography (eluent: 2: 1 petroleum ether and ethyl acetate) to afford compound **2** (1.91 g, 5.75 mmol) as a white solid. M. p. 103-107 ℃. ^{1}H NMR (400 MHz, $CDCl_3$): δ 1.54-1.57 (m, 4H, CH_2CH_2), 1.81-1.85 (m, 2H, CH_2), 1.87-1.90 (m, 2H, CH_2), 4.11 (t, J = 8 Hz, 2H, OCH_2), 4.35 (t, J = 8 Hz, 2H, OCH_2), 7.25-7.28 (m, 1H, Py-H), 7.42-7.45 (m, 2H, Ar-H), 7.54-7.56 (m, 1H, Ar-H), 7.94 (d, J = 8 Hz, 1H, Py-H), 8.04 (d, J = 8 Hz, 2H, Ar-H), 8.41-8.42 (m, 1H, Py-H), 9.99 (s, 1H, CHO). ^{13}C NMR (125 MHz, $CDCl_3$): δ 25.8, 26.0, 28.8, 29.1, 65.0, 68.9, 120.7, 123.6, 128.5, 129.7, 130.6, 133.1, 138.9, 146.3, 158.7, 166.8, 192.2. HRMS (ESI) for $C_{19}H_{22}NO_4$, *m/z*, $[M + H]^+$, calculated: 328.1543, found: 328.1537.

Synthesis of 3. Compound **2** (1.9 g, 5.75 mmol) was dissolved in MeOH (20 mL) and then $NaBH_4$ (0.66g, 17.4 mmol) was added to the solution. After stirred 2 h, water was added to the mixture. Then, dichloromethane (DCM, 30 mL) was add to the mixture. The organic layer was collected. Water layer was washed with DCM for 3 times. DCM portion was dried with magnesium sulfate anhydrous and concentrated under reduced pressure. The crude product was further purified by flash column chromatography (eluent: 10: 1, DCM/MeOH) to afford **3** as a colorless oil (1.92 g, 5.76 mmol). ^{1}H NMR (400 MHz, $CDCl_3$): δ 1.54-1.80 (m, 4H, CH_2CH_2), 1.82-1.86 (m, 4H, $2CH_2$), 2.39 (br, 1H, OH), 4.01 (t, *J* = 8 Hz, 2H, OCH_2), 4.34 (t, *J* = 8 Hz, 2H, OCH_2), 4.72 (s, 2H, Py-CH_2), 7.19-7.26 (m, 2H, Py-H), 7.41-7.45 (m, 2H, Ar-H), 7.53-7.57 (m, 1H, Ar-H), 8.03 (d, *J* = 4 Hz, 2H, Ar-H), 8.23 (s, 1H, Py-H). ^{13}C NMR (125 MHz, $CDCl_3$): δ 25.9, 26.0, 28.8, 29.3, 64.2, 65.1, 68.6, 121.3, 122.7, 128.5, 129.7, 130.6, 133.1, 136.3, 151.3, 154.6, 166.9. HRMS (ESI) for $C_{19}H_{24}NO_4$, *m/z*, $[M + H]^+$, calculated: 330.1699, found: 330.1694.

Synthesis of 4. Compound **1** (1.9 g, 5.75 mmol) and **3** (1.63 g, 5.78 mmol) were dissolved in THF (20 mL). Then, DIAD (1.8 ml, 7.47 mmol) and PPh_3 (2.05 g, 7.47 mmol) were added to the solution. The mixture was stirred for 12 h and purified by flash column chromatography (eluent: DCM). Compound **4** (3.15 g, 5.32 mmol) was afford as a colorless oil. ^{1}H NMR (400 MHz, $CDCl_3$): δ 1.36 (s, 20H, CH_3, CH_2), 1.80-1.84 (m, 4H, CH_2CH_2), 2.00-2.03 (m, 2H, CH_2), 4.03 (t, *J* = 8 Hz, 2H, OCH_2), 4.34 (t, *J* = 8 Hz, 2H, OCH_2), 5.19 (s, 2H, CH_2), 7.04 (d, *J* = 1.2 Hz, 2H, Ar-H), 7.20-7.23 (m, 1H, Py-H), 7.36-7.55 (m, 6H, Py-H, Ar-H), 8.03 (d, *J* = 1.2 Hz, 2H, Ar-H), 8.28-8.29 (m, 1H, Py-H). ^{13}C NMR (125 MHz, $CDCl_3$): δ 25.9, 26.0, 29.2, 31.7, 32.9, 35.1, 65.0, 68.5, 70.7, 115.2, 121.1, 121.5, 122.1, 122.3, 128.5, 128.6, 129.7, 130.6, 133.0, 135.6, 137.3, 140.3, 149.1, 151.2, 154.8, 158.0, 166.8. HRMS (ESI) for $C_{39}H_{48}NO_4$, *m/z*, $[M + H]^+$, calculated: 594.3577, found: 594.3568.

Synthesis of 5. Compound **4** (3 g, 5.06 mmol) was dissolved in MeOH and DCM (2/1, v/v), and then KOH (0.85 g, 15.18 mmol) was added to the solution. After stirred overnight, the crude product was further purified by flash column chromatography (eluent: 40: 1, DCM/MeOH) to afford **5** as a colorless oil (1.97 g, 4.05 mmol). ^{1}H NMR (400 MHz, $CDCl_3$): δ 1.36 (s, 18H, CH_3), 1.44-1.64 (m, 6H, $CH_2CH_2CH_2$), 1.78-1.84 (m, 2H, CH_2), 3.67 (t, J = 8 Hz, 2H, OCH_2), 4.01 (t, J = 1.2 Hz, 2H, OCH_2), 5.18 (s, 2H, CH_2), 7.04 (d, J = 1.2 Hz, 2H, Ar-H), 7.19-7.23 (m, 1H, Py-H), 7.36-7.39 (m, 3H, Ar-H), 7.43-7.46 (m, 1H, Py-H), 7.50 (d, J = 8 Hz, 2H, Ar-H), 8.28 (d, J = 4 Hz, 1H, Py-H). ^{13}C NMR (125 MHz, $CDCl_3$): δ 25.7, 26.0, 29.3, 31.7, 32.8, 35.1, 63.0, 68.5, 70.7, 115.2, 121.1, 121.5, 122.0, 122.3, 128.6, 135.6, 137.4, 140.3, 149.0, 151.2, 154.8, 158.0. HRMS (ESI) for $C_{32}H_{44}NO_3$, *m/z*, $[M + H]^+$, calculated: 490.3315, found: 490.3307.

Synthesis of 6. Compound **5** (1 g, 2.04 mmol) was dissolved in DCM (4 mL) and the solution of HCl in water (2 M, 2ml) was added. The mixture was stirred overnight to fully exchange ions. Then, the DCM layer was separated and was with water 2 times. The DCM layer was dried with magnesium sulfate anhydrous and concentrated under reduced pressure to afford **6** (2.73 g, 2.02 mmol) as a yellowish oil. ^{1}H NMR (400 MHz, CD_3CN): δ 0.02 (s, 18H, CH_3), 0.07-0.22 (m, 6H, $CH_2CH_2CH_2$), 0.51-0.54 (m, 2H, CH_2), 2.20 (t, J = 5 Hz, 2H, OCH_2), 2.89 (t, J = 10 Hz, 2H, OCH_2), 4.07 (s, 2H, CH_2), 5.78-5.80 (m, 2H, Ar-H), 5.99-6.00 (m, 2H, Ar-H), 6.04-6.05 (m, 1H, Ar-H), 6.19-6.23 (m, 14H, Ar-H), 6.70-6.71 (m, 1H, Py-H), 6.83-6.85 (m, 1H, Py-H), 7.15 (d, J = 5 Hz, 1H, Py-H). ^{13}C NMR (125 MHz, CD_3CN): δ 26.6, 26.7, 29.7, 32.0, 33.7, 36.0, 62.9, 66.3, 71.9, 116.6, 122.5, 122.7, 124.7, 126.9, 127.7, 129.1, 129.3, 129.9-130.7 (m), 131.7, 134.8, 136.0, 134.4-144.7 (q, $^1J_{CF}$ = 462 Hz), 152.8, 157.9, 158.8, 162.4-163.6 (q, $^1J_{CB}$ = 50 Hz). HRMS (ESI) for $C_{32}H_{44}NO_3$, *m/z*, $[M + H]^+$, calculated: 490.3311, found: 490.3315.

Synthesis of R. Compound **6** (0.22 g, 0.16 mmol) and **H** (50 mg, 0.05 mmol) were charged to dried DCM (2 ml) in a Schlenk tube and stirred for 6 h. Then, *p*-tritylphenylisocyanate (130 mg, 0.36 mmol) and catalytic amount of DBTDL (dibutyltin dilaurate) were added to the solution and keep stirring for overnight. After the reaction finished, a large number of white precipitation produced. The precipitates was filter out and the solution was concentrated under reduced pressure. The mixture were purified by flash column chromatography (eluent: 1: 2, PE/DCM) to afford **R** as a white powder (37 mg, 0.02 mmol). M. p. 161.2-164.9. It was noteworthy that, maybe due to the relative weak of combining ability between **H** and protonated pyridium site and relative weak alkalinity of pyridium, the obtained rotaxane was deprotonated state. ^{1}H NMR (300 MHz, CD_2Cl_2): δ -1.63 (br. 4H, CH_2CH_2), -0.54 (br. 2H, CH_2), -0.29 (br. 2H, CH_2), 1.52 (s, 18H, CH_3), 2.42 (m, 2H, OCH_2), 3.06 (m, 2H, OCH_2), 3.59 (s, 9H, OCH_3), 3.66 (s, 6H, $ArCH_2Ar$), 3.83 (s, 9H, OCH_3), 5.03 (s, 3H, CH), 5.11 (s, 3H, CH), 5.35 (s, 2H, CH_2), 6.62 (s, 3H, Ar-H), 6.81 (s, 3H, Ar-H), 6.81-6.90 (m, 6H, Ar-H), 7.16-7.40 (m, 30H, Ar-H, Py-H), 7.56-7.71 (m, 11H, Ar-H), 8.20 (s, 1H, Py-H). ^{13}C NMR (125 MHz, CD_2Cl_2): δ 26.0, 26.9, 27.7, 28.8, 30.2, 31.8, 35.3, 46.6, 56.0, 56.7, 65.1, 68.2, 107.6, 108.3, 115.6, 117.7, 121.5, 121.7, 122.1, 123.0, 123.4, 125.2, 125.8, 126.5, 127.1, 127.3, 128.1, 128.9, 130.4, 131.5, 132.5, 135.3, 137.6, 137.8, 144.9, 145.0, 146.9, 147.0, 147.5, 151.8, 154.4, 154.5, 158.5. HRMS (ESI) for $C_{127}H_{117}N_2O_{10}$, *m/z*, $[M + H]^+$, calculated: 1830.8713, found: 1830.8725.

3. Copies of NMR spectra of new compounds

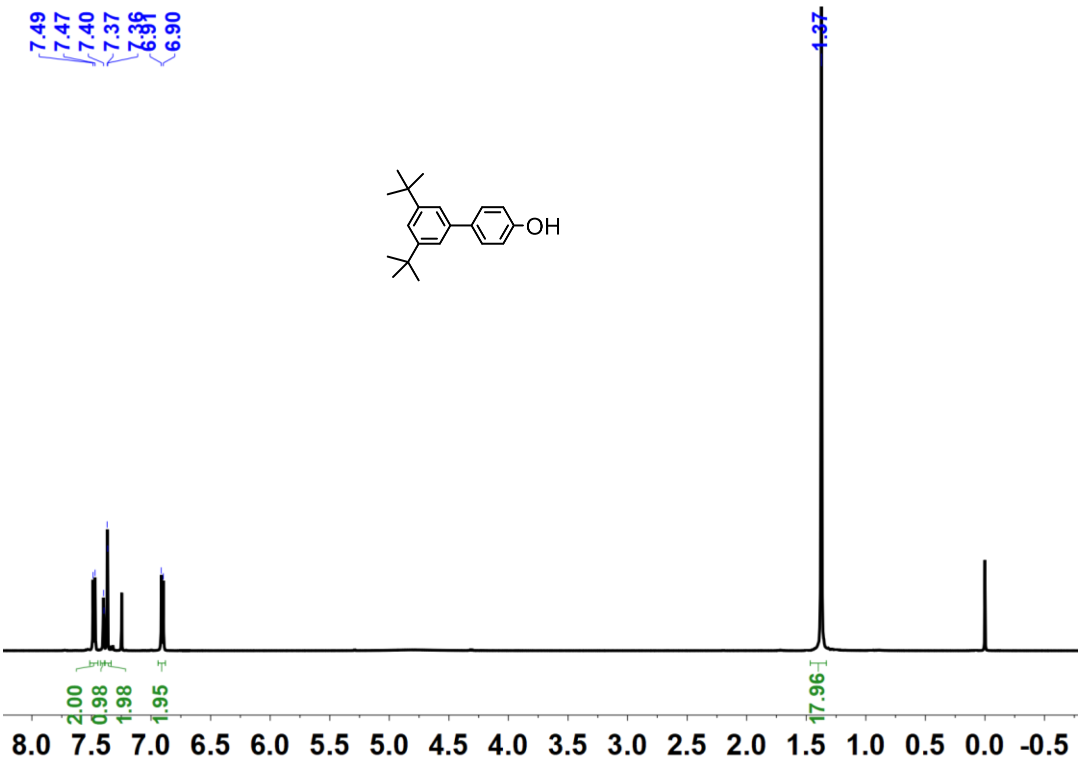

Fig. S1 ^{1}H NMR (500 MHz, $CDCl_3$, 298 K) of **1**.

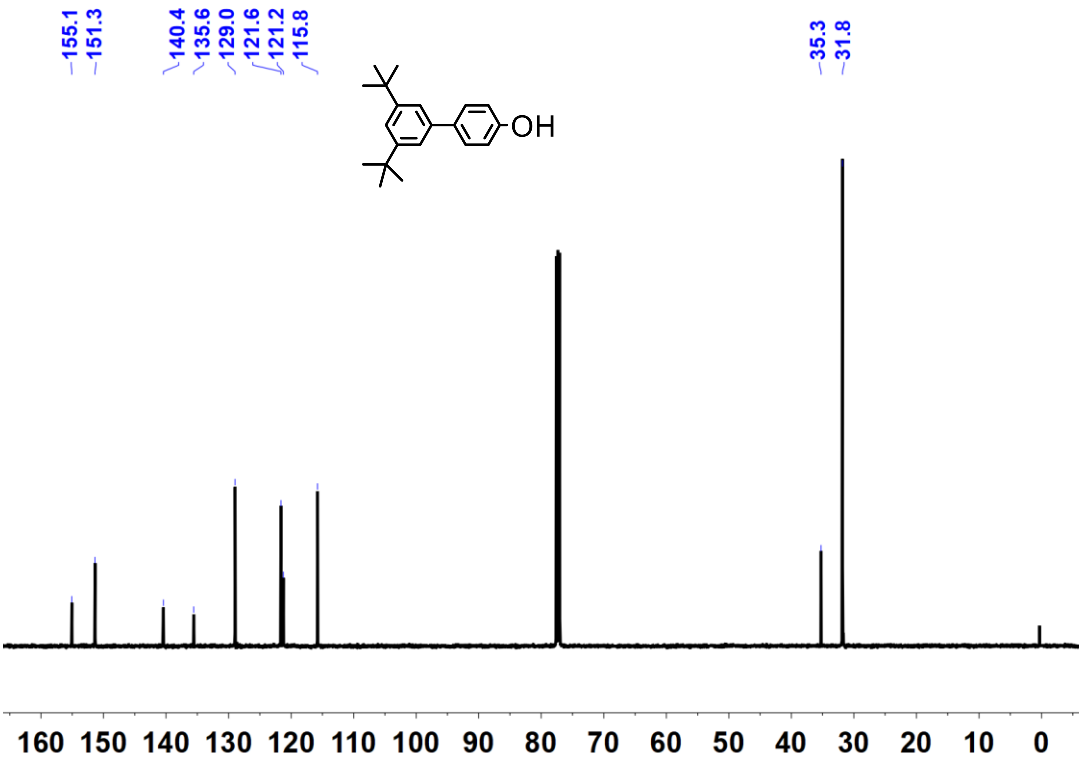

Fig. S2 ^{13}C NMR (125 MHz, $CDCl_3$, 298 K) of **1**.

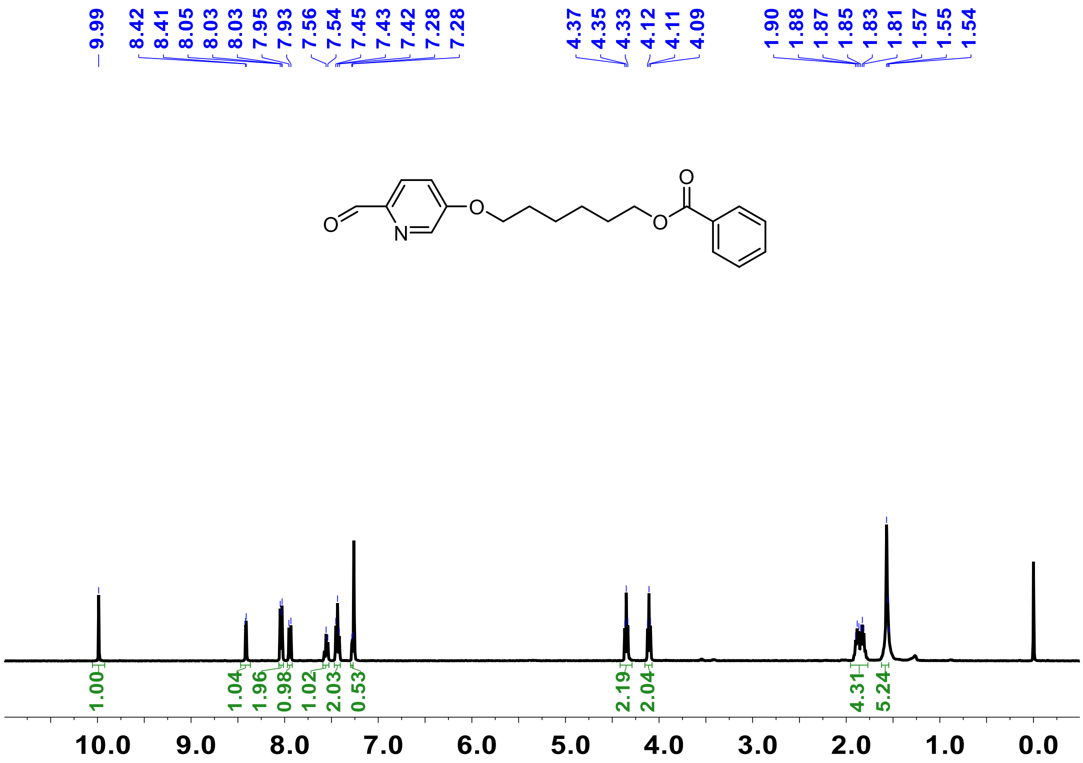

Fig. S3 ^{1}H NMR (400 MHz, $CDCl_3$, 298 K) of **2**.

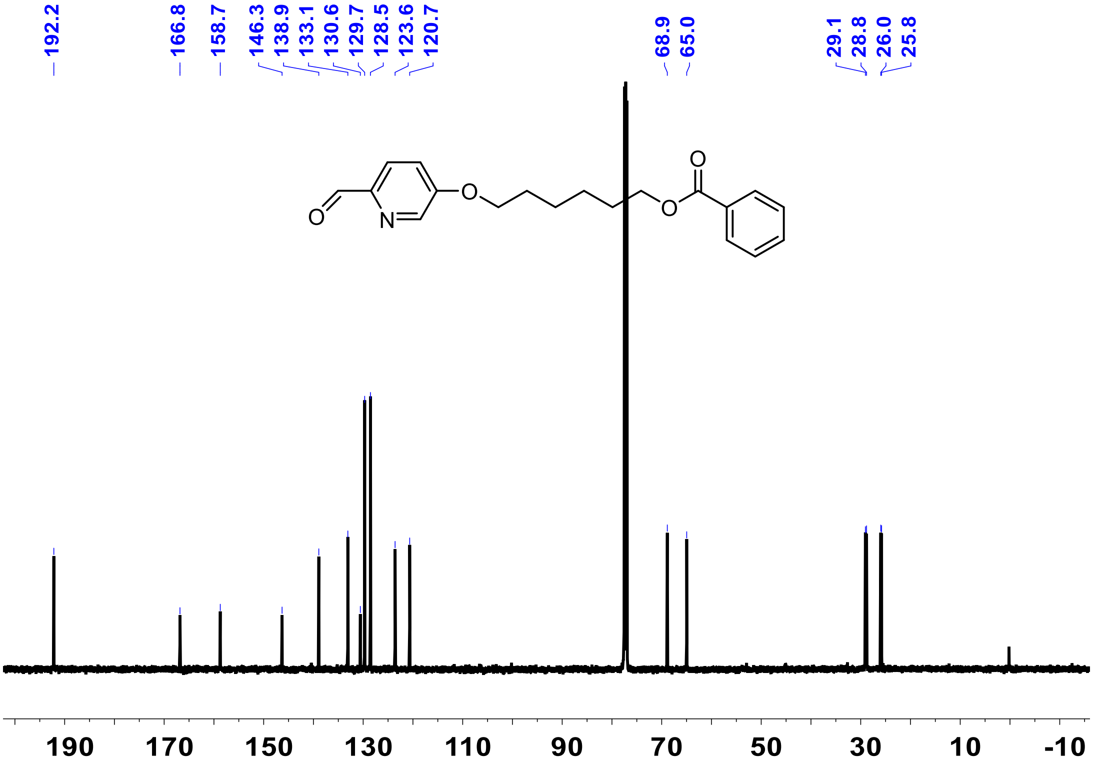

Fig. S4 ^{13}C NMR (125 MHz, $CDCl_3$, 298 K) of **2**.

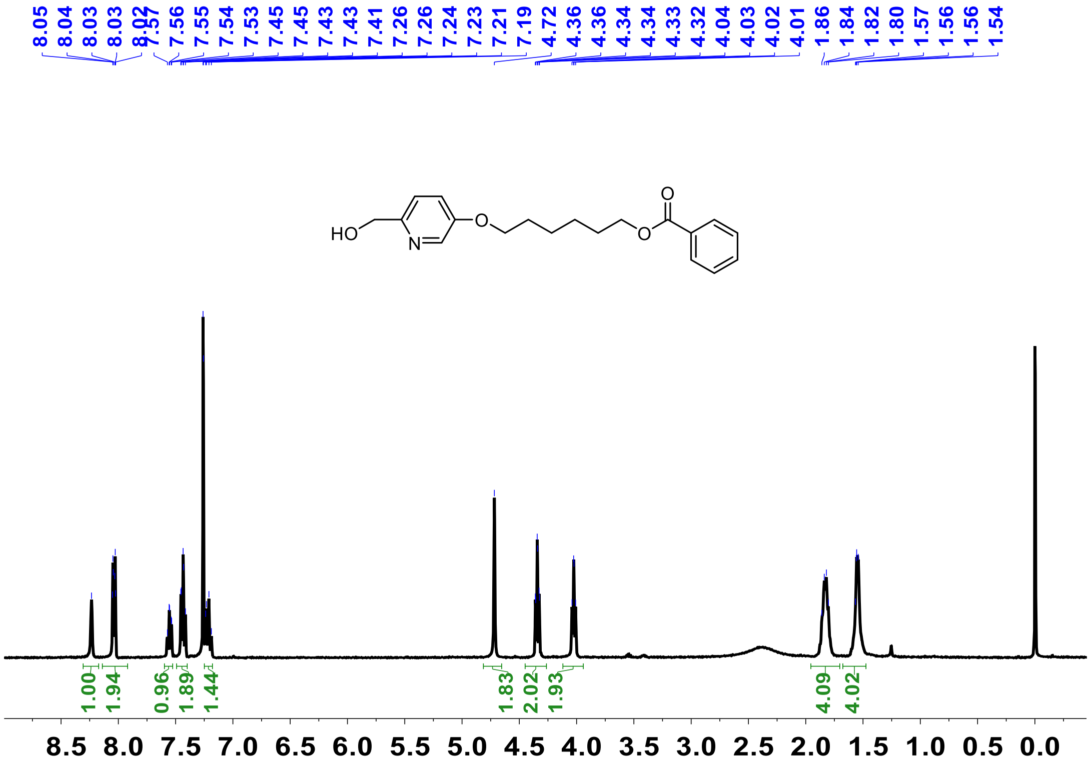

Fig. S5 ^{1}H NMR (400 MHz, $CDCl_3$, 298 K) of **3**.

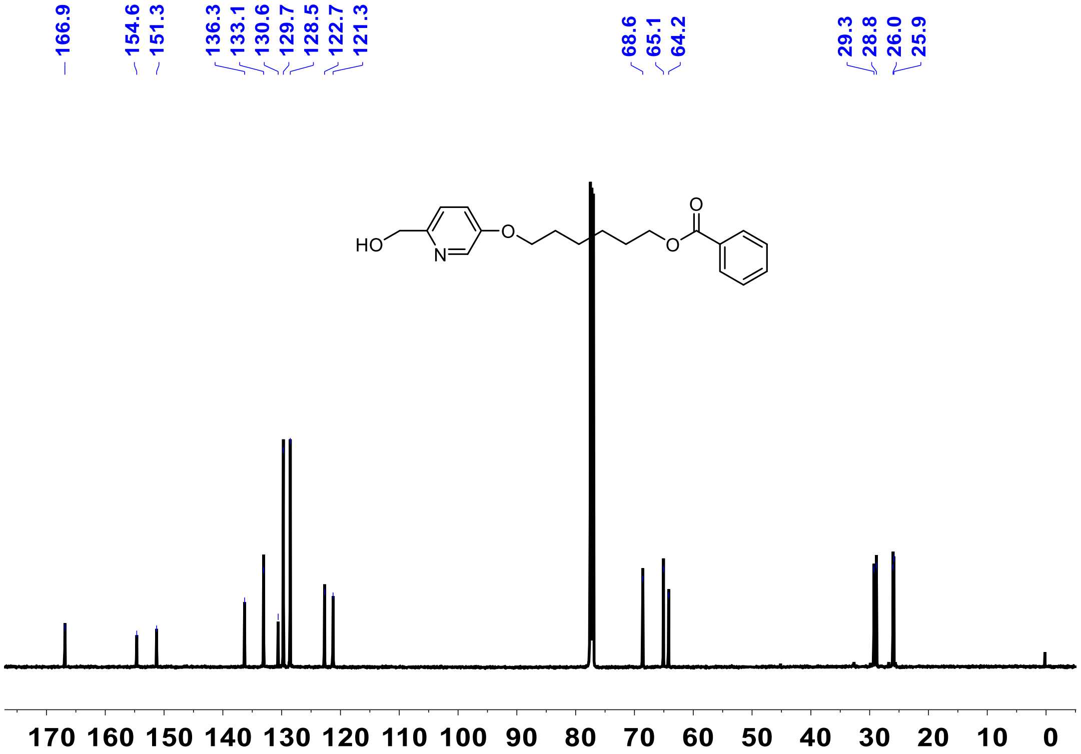

Fig. S6 ^{13}C NMR (125 MHz, $CDCl_3$, 298 K) of **3**.

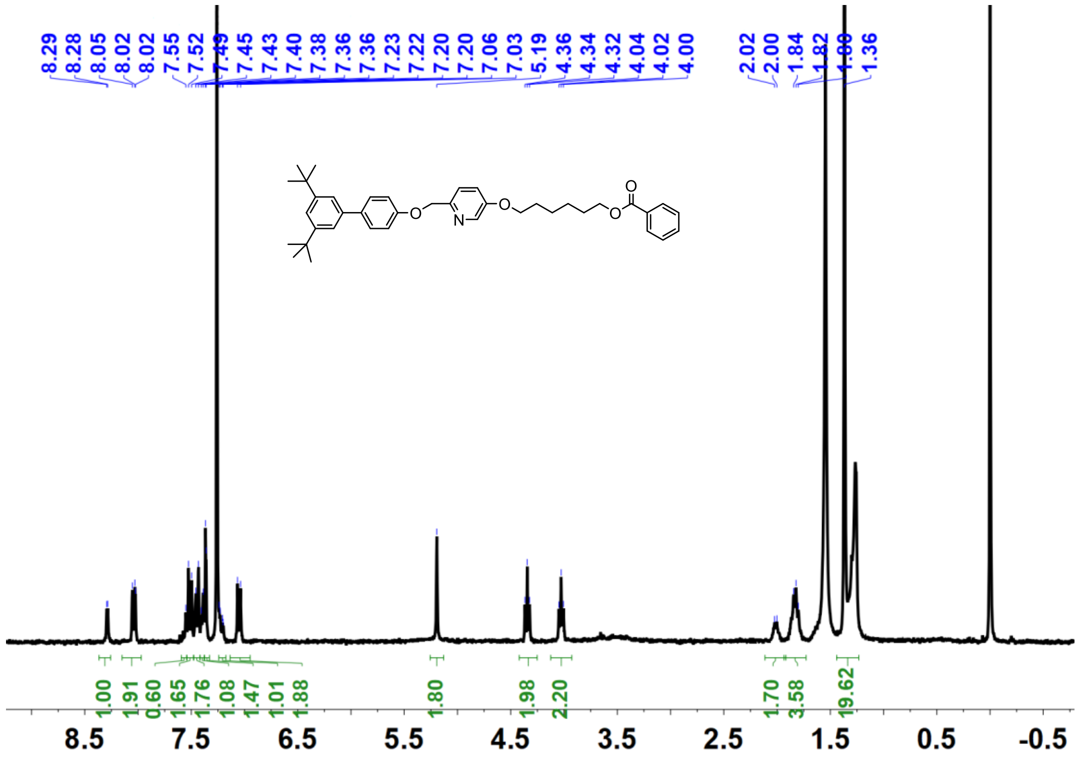

Fig. S7 ^{1}H NMR (300 MHz, $CDCl_3$, 298 K) of **4**.

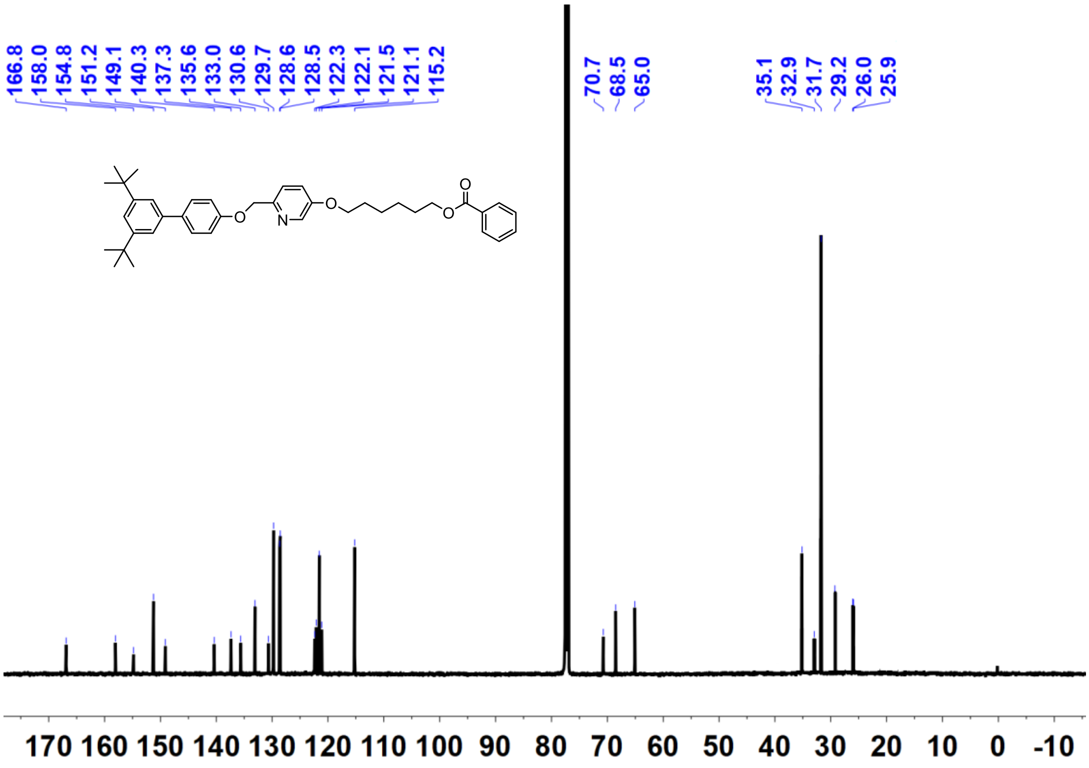

Fig. S8 ^{13}C NMR (125 MHz, $CDCl_3$, 298 K) of **4**.

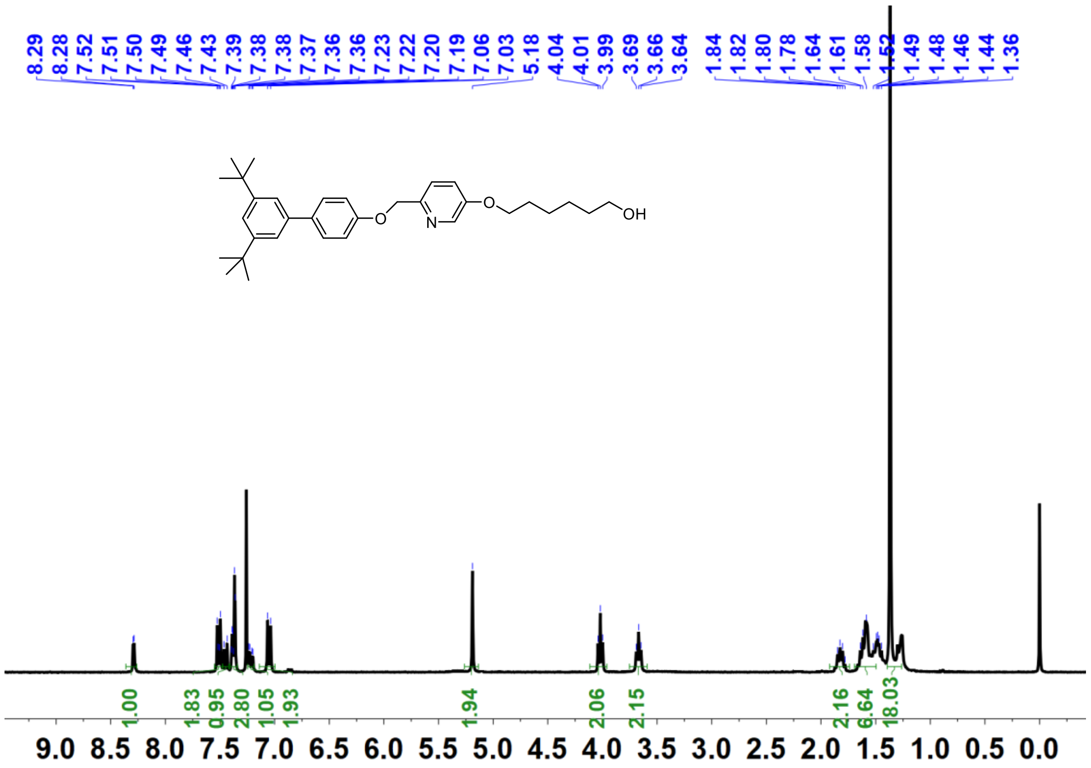

Fig. S9 ^{1}H NMR (300 MHz, $CDCl_3$, 298 K) of **5**.

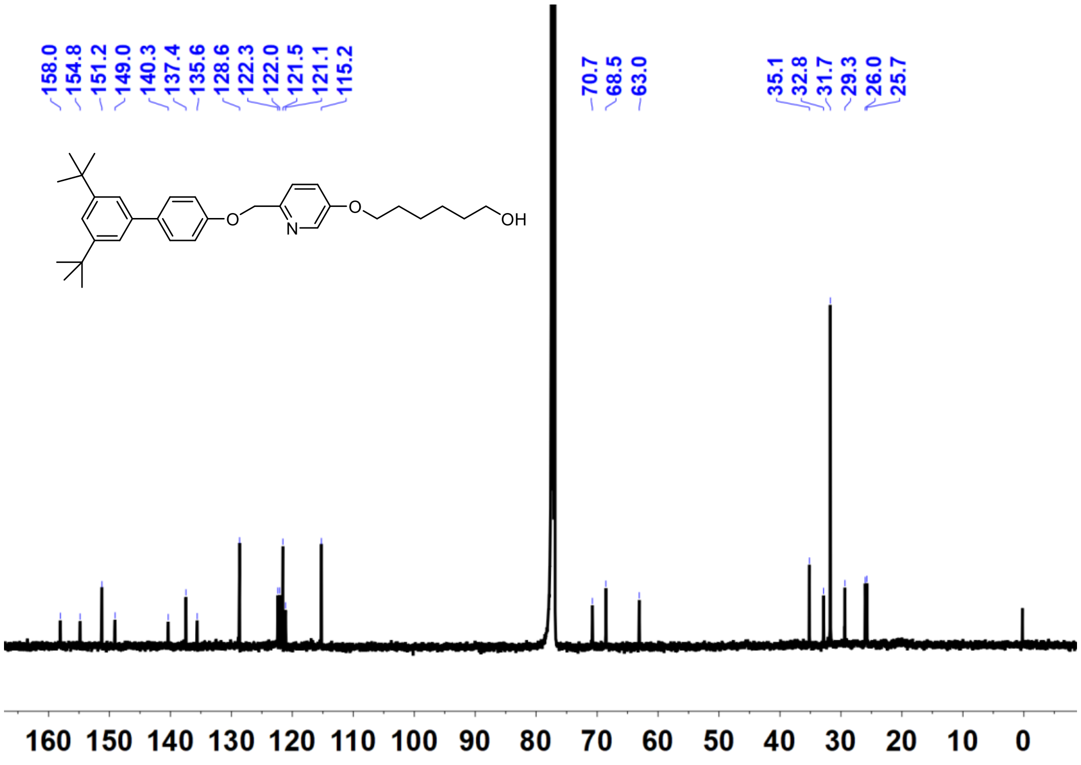

Fig. S10 ^{13}C NMR (125 MHz, $CDCl_3$, 298 K) of **5**.

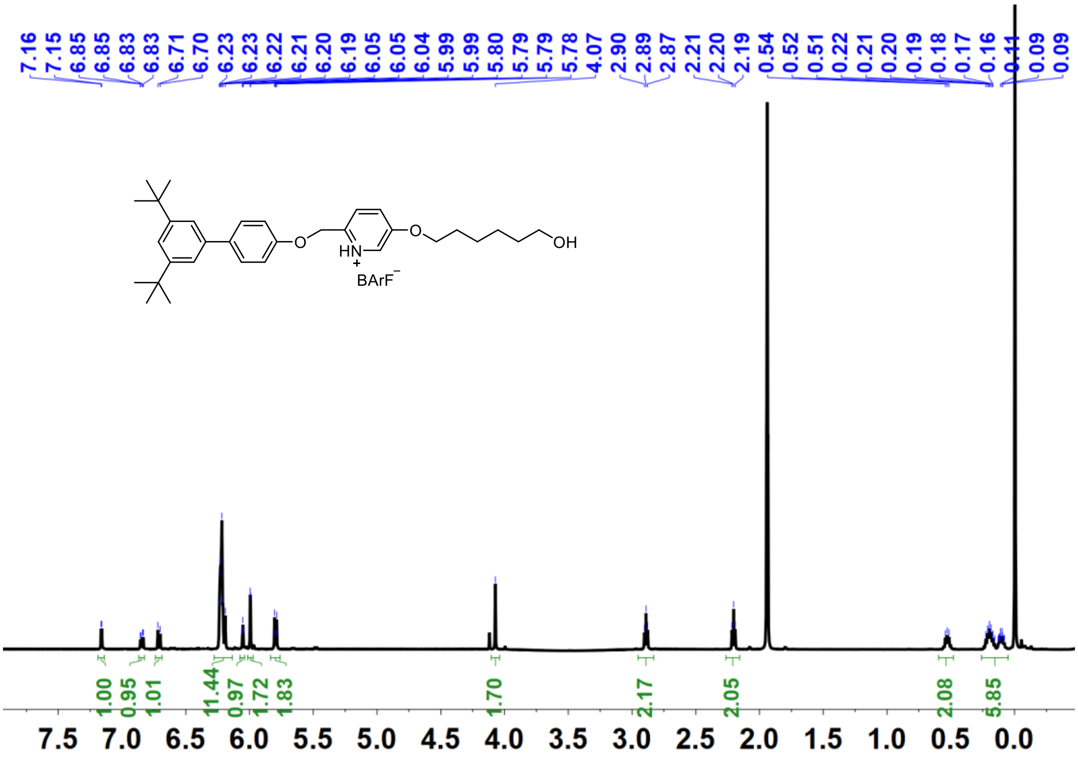

Fig. S11 ^{1}H NMR (500 MHz, CD_3CN, 298 K) of **6**.

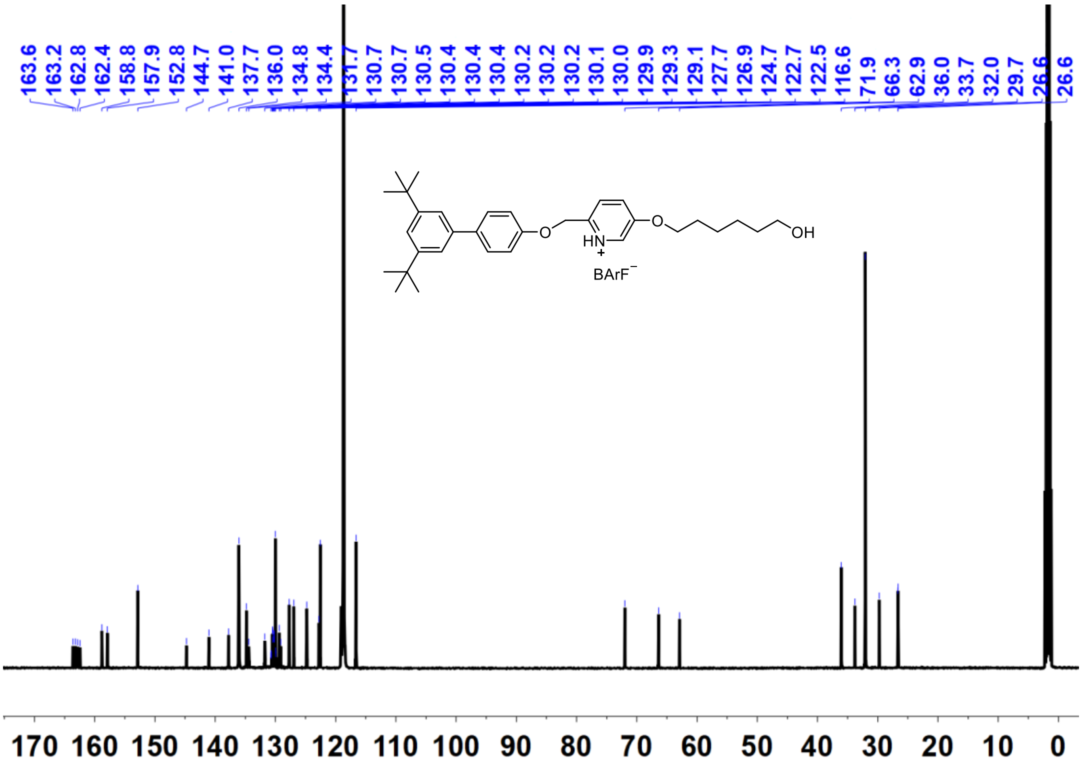

Fig. S12 ^{13}C NMR (125 MHz, CD_3CN, 298 K) of **6**.

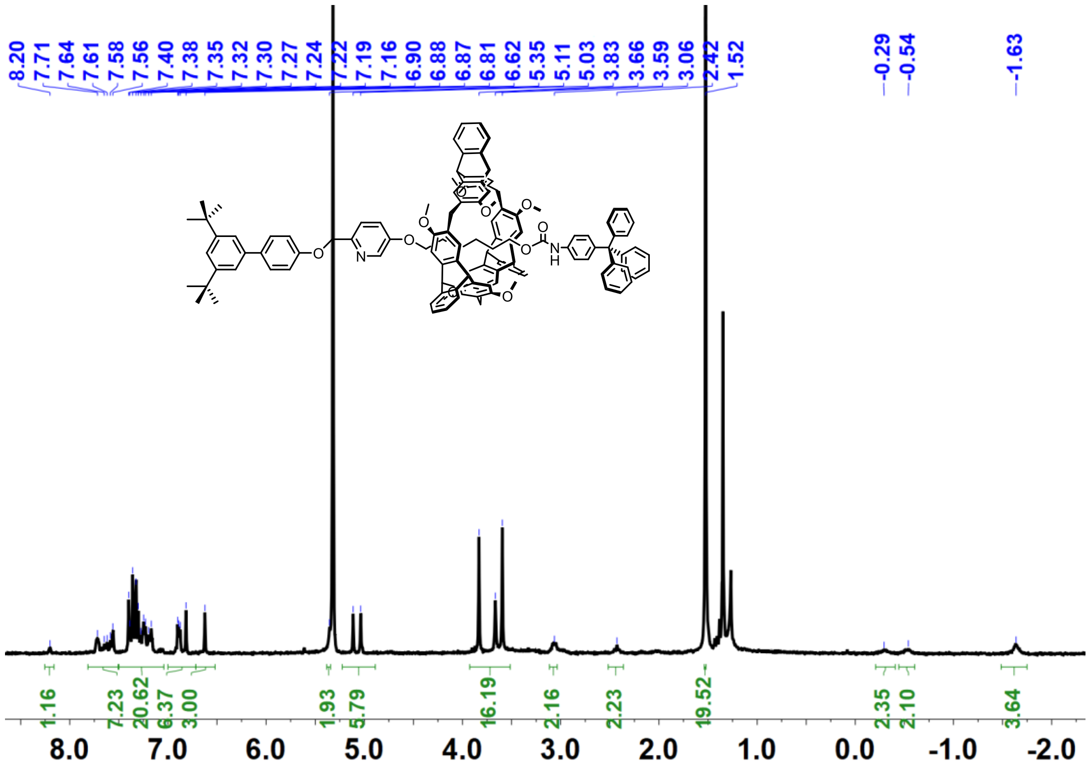

Fig. S13 ^{1}H NMR (300 MHz, CD_2Cl_2, 298 K), of **R**.

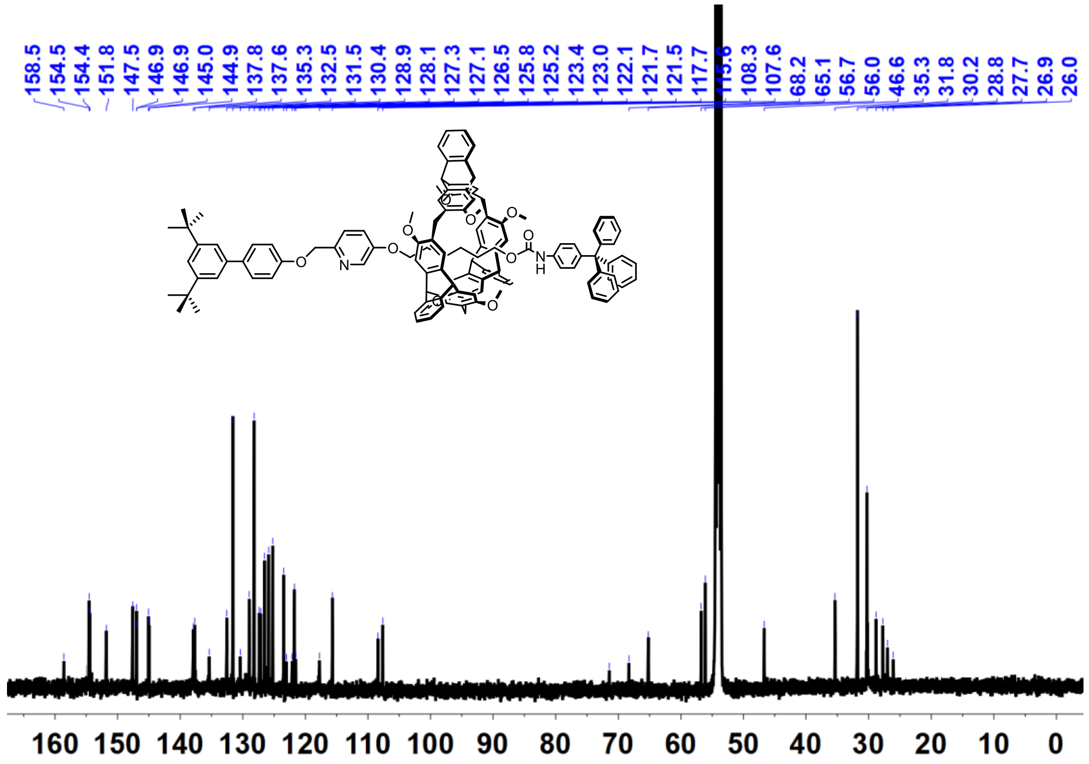

Fig. S14 ^{13}C NMR (125 MHz, CD_2Cl_2, 298 K) of **R**.

4. Acid-base controlled motion of the [2] rotaxane R

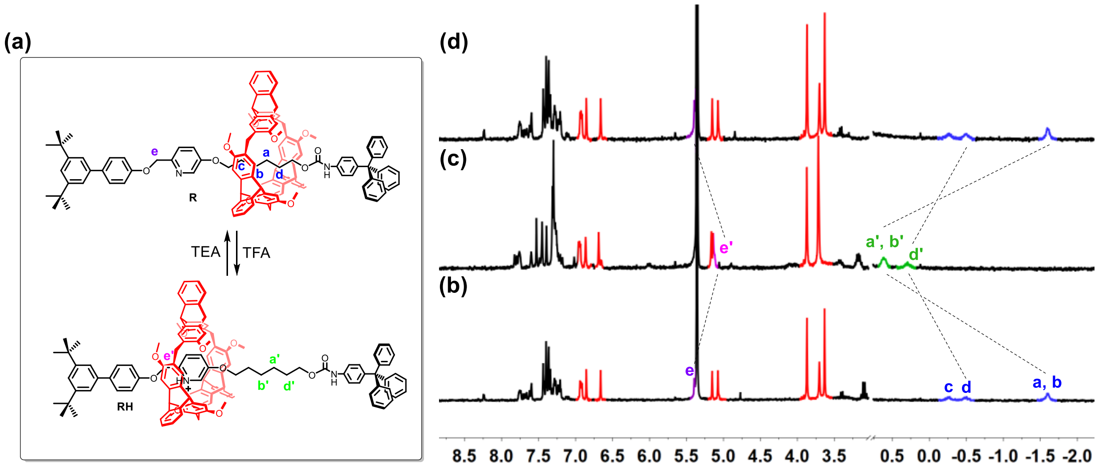

Fig. S15 (a) the acid-base switched motion of **R** and ^{1}H NMR (300 MHz, CD_2Cl_2, 298 K) of (b) **R**, (c) **RH** (**R** + 4 equiv TFA) and (d) c + 5 equiv TEA. (The volume of the solution is 0.5 mL, and the concentration of **R** is 2 mM.)

5. The dynamics of the oxidation reaction controlled motion of R powered by BTAIB

Experimental method. The rotaxane **R** was dissolved in 0.5 mL CD_2Cl_2 (c = 2 mM). And then, 0.5 equiv TEMPO and excess (25 equiv) *i*-PrOH was added to the solution. When 2 equiv BTAIB was added, the ^{1}H NMR data was recorded immediately as the t = 0 h point. Then, the data were recorded at t = 0.5 h, 1.0 h, 2.0 h, 4.5 h, 6.0 h and 8.0 h. According to the following equation:

$$x = \frac{\delta - \delta''}{\delta' - \delta''}$$

Where x is the relative content of **R**. The δ value is the chemical shifts of H_a or H_b in different time. The δ' value is the chemical shifts of H_a or H_b in pure **R** (t = 0 h). And δ'' is the chemical shifts of H_a or H_b in pure **RH**. The relative contents of **R** and **RH** in different times were calculated and showed in Fig. S17.

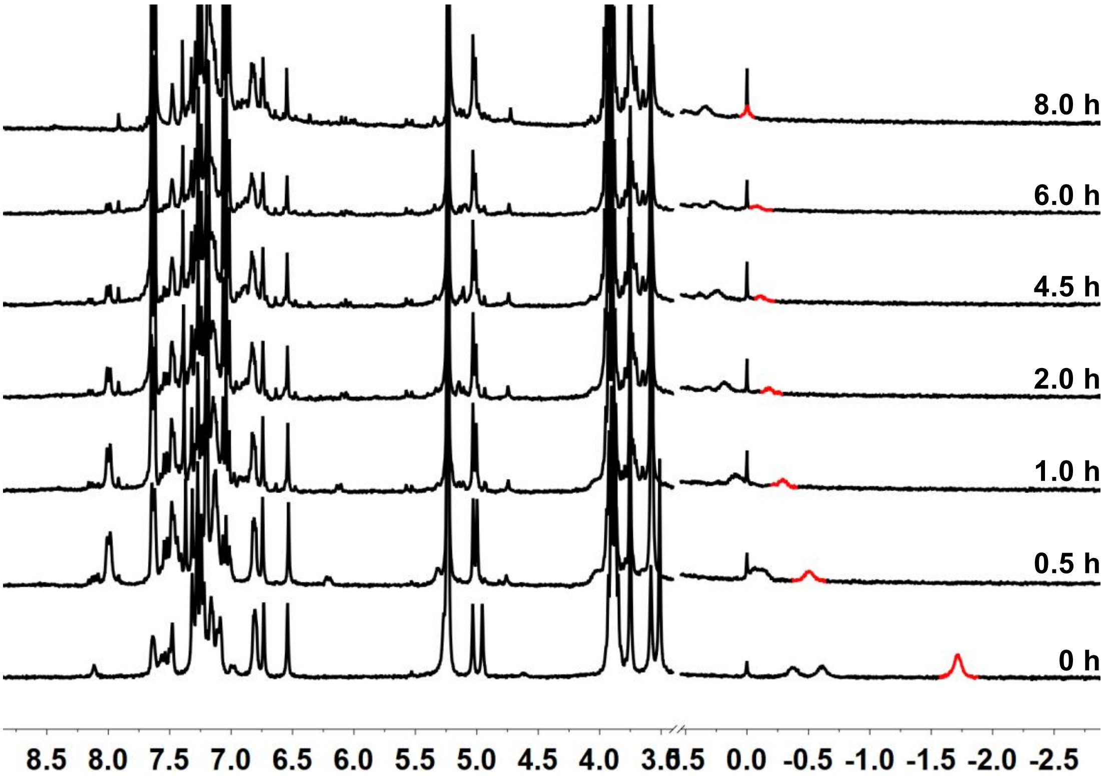

Fig. S16 ^{1}H NMR of the oxidation reaction controlled motion of **R** in different time.

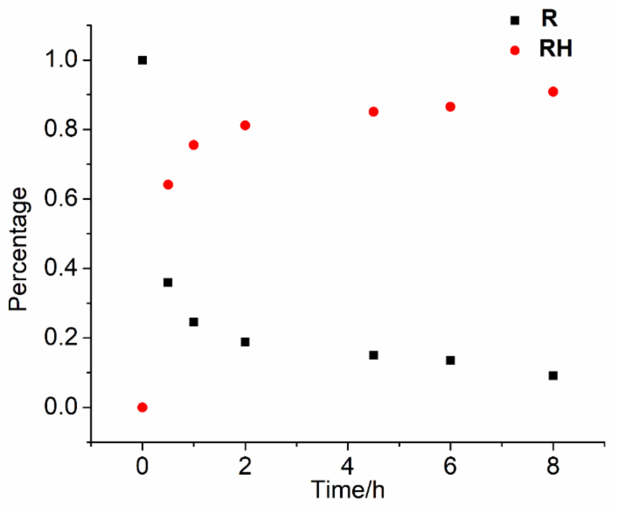

Fig. S17 Percentage of **R** and **RH** in the course of the catalytic oxidation reaction in different time.

6. The dynamic of the mechanical motion of R with different amount of TEMPO

Experimental method. The rotaxane **R** was dissolved in 0.5 mL CD_2Cl_2 (c = 2 mM). TFA was added to prepare **RH** in solution after adding 0.5 equiv or 1.0 equiv TEMPO and excess *i*-PrOH. Then, the chemical fuel PhIO (2 equiv of TFA) was added to the solution to trigger the normal operation of the whole system. The NMR data were recorded in different time.

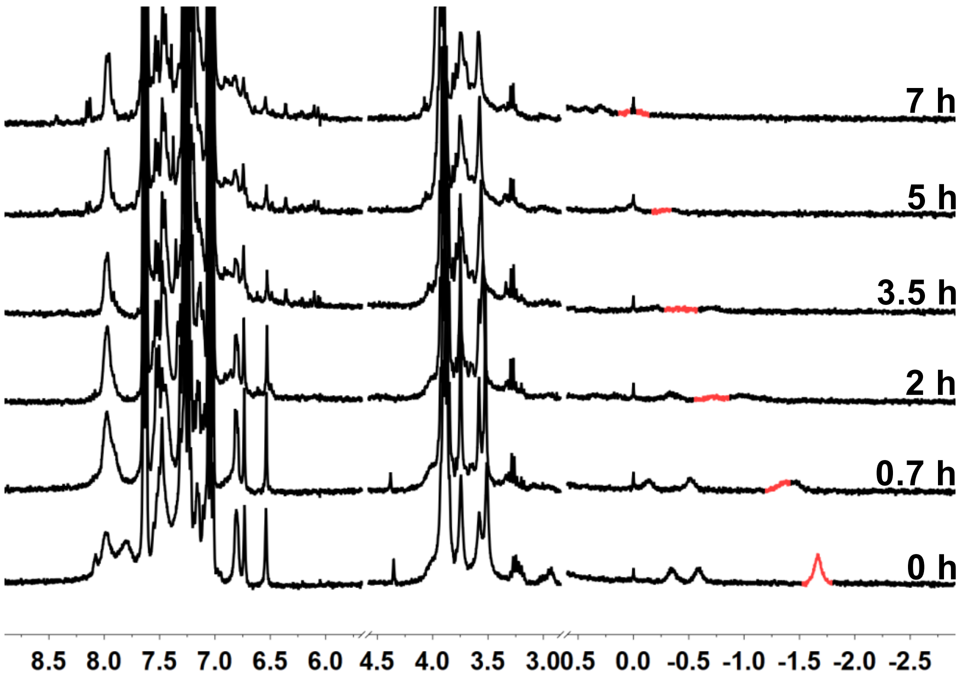

Fig. S18 ^{1}H NMR of the motion of **R** in different time (0.5 equiv tempo).

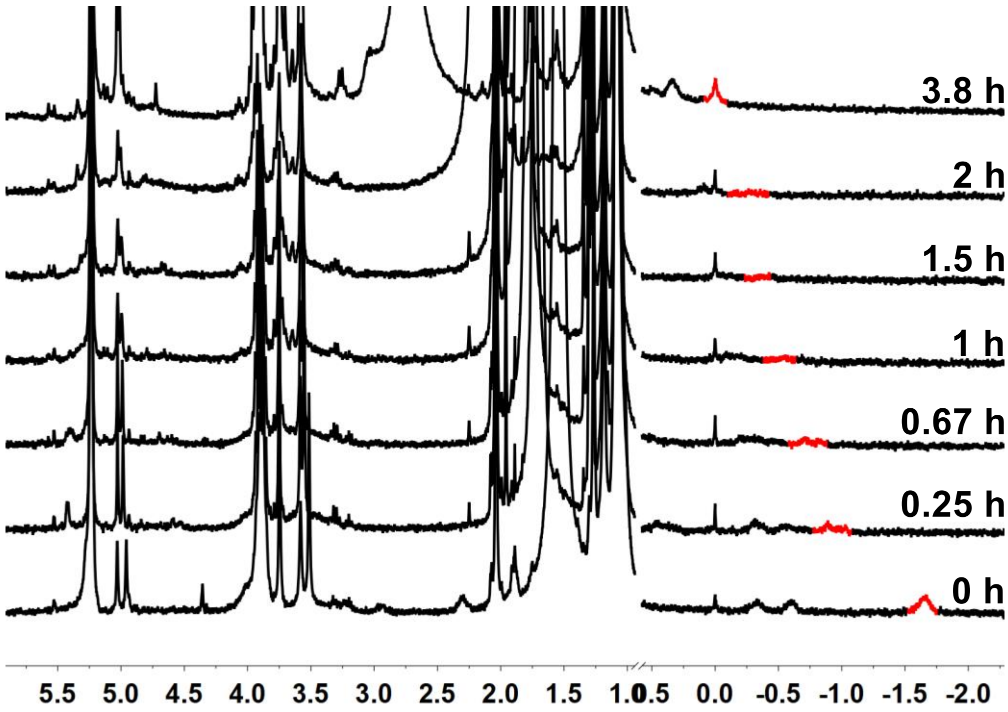

Fig. S19 ^{1}H NMR of the motion of **R** in different time (1.0 equiv tempo).

7. Repeated Chemically Fueled motion

Experimental method. The mother solution was prepared according to the method mentioned above. Then the first port of PhIO was added to the mother solution and the NMR date of the whole process were recorded. After this process, the second port of PhIO was added to the solution and the NMR data of the process were recorded. Four repeated chemically fuelled motion were recorded.

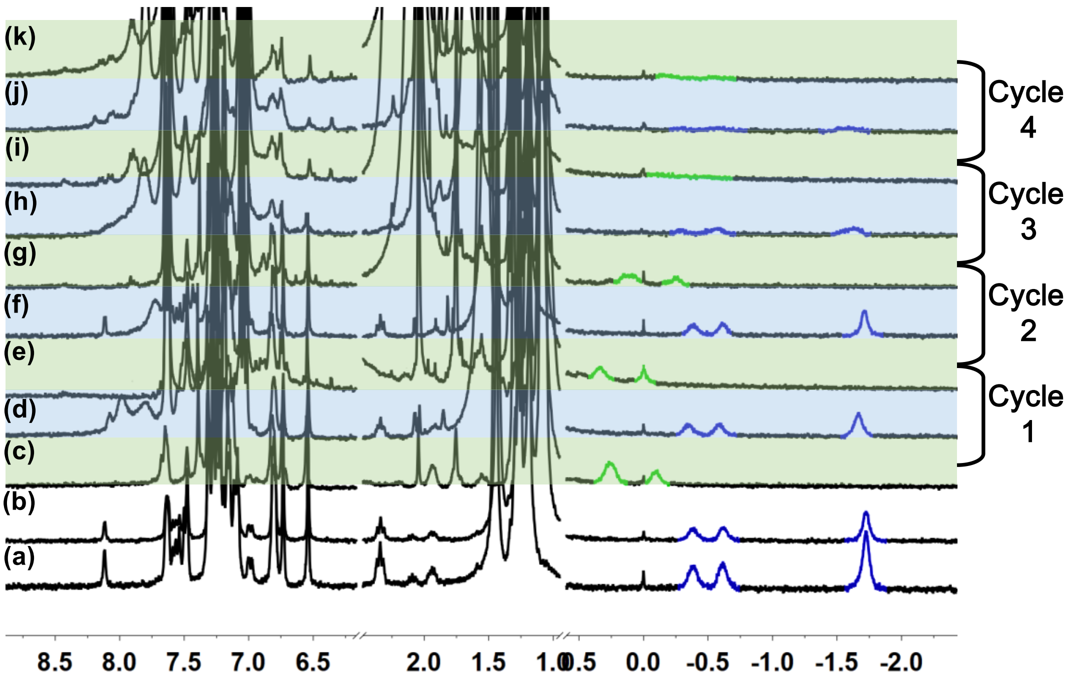

Fig. S20 ^{1}H NMR (300 MHz, CD_2Cl_2, 298 K) of (a) **R**, (b) **R** + 0.5 equiv tempo + 25 equiv iPrOH and four cycles of motion.
